# Nationwide Investigation of the Pyrethroid Susceptibility of Mosquito Larvae Collected from Used Tires in Vietnam

**DOI:** 10.1371/journal.pntd.0000391

**Published:** 2009-03-10

**Authors:** Hitoshi Kawada, Yukiko Higa, Yen T. Nguyen, Son H. Tran, Hoa T. Nguyen, Masahiro Takagi

**Affiliations:** 1 Department of Vector Ecology & Environment, Institute of Tropical Medicine, Nagasaki University, Nagasaki, Japan; 2 National Institute of Hygiene and Epidemiology, Hanoi, Vietnam; George Washington University, United States of America

## Abstract

Pyrethroid resistance is envisioned to be a major problem for the vector control program since, at present, there are no suitable chemical substitutes for pyrethroids. Cross-resistance to knockdown agents, which are mainly used in mosquito coils and related products as spatial repellents, is the most serious concern. Since cross-resistance is a global phenomenon, we have started to monitor the distribution of mosquito resistance to pyrethroids. The first pilot study was carried out in Vietnam. We periodically drove along the national road from the north end to the Mekong Delta in Vietnam and collected mosquito larvae from used tires. Simplified susceptibility tests were performed using the fourth instar larvae of *Aedes aegypti*, *Aedes albopictus*, and *Culex quinquefasciatus*. Compared with the other species, *Ae. aegypti* demonstrated the most prominent reduction in susceptibility. For *Ae. aegypti*, significant increases in the susceptibility indices with a decrease in the latitude of collection points were observed, indicating that the susceptibility of *Ae. aegypti* against *d*-allethrin was lower in the southern part, including mountainous areas, as compared to that in the northern part of Vietnam. There was a significant correlation between the susceptibility indices in *Ae. aegypti* and the sum of annual pyrethroid use for malaria control (1998–2002). This might explain that the use of pyrethroids as residual treatment inside houses and pyrethroid-impregnated bed nets for malaria control is attributable to low pyrethroid susceptibility in *Ae. aegypti*. Such insecticide treatment appeared to have been intensively administered in the interior and along the periphery of human habitation areas where, incidentally, the breeding and resting sites of *Ae. aegypti* are located. This might account for the strong selection pressure toward *Ae. aegypti* and not *Ae. albopictus*.

## Author Summary

Pyrethroid is one of the most successful insecticidal chemicals for controlling insect pests and vectors of household and public health importance. However, extensive treatment of photo-stable and highly effective pyrethroids sometimes causes resistance to insect populations. Resistance to pyrethroids belonging to the knockdown agent groups that have been used as the spatial repellents, such as mosquito coils and related products, is the most serious concern. Since this is a global phenomenon, we have started to monitor the distribution of mosquito resistance to pyrethroids and the first pilot study was carried out in Vietnam. Our study concluded that *Aedes aegypti*, the most important vector of dengue and dengue haemorrhagic fever, demonstrated the prominent reduction in susceptibility against *d*-allethrin, one of the most popular pyrethroids for spatial repellents. The extensive treatment of photo-stable pyrethroids for malaria control seemed to be one of the attributable factors. In order to effectively manage pyrethroid resistance, the establishment of a feasible insecticide management system and a regular monitoring system of pyrethroid susceptibility will be essential.

## Introduction

One of the most successful events in the development of pesticide chemicals was the discovery of pyrethrum and the successful synthesis of pyrethroids. For example, allethrin [1], a classical synthetic pyrethroid, continues to be used for preventing mosquito bites without any toxicological and operational problems. There are two main groups of pyrethroids: one possessing high knockdown activity but low killing activity and the other possessing high killing activity. The pyrethroids in the former group, such as *d*-allethrin, are aptly labeled as knockdown agents, and those in the latter group, as killing agents. Generally, the pyrethroids belonging to the latter group exhibit high photostability that enables their outdoor use, for example, as agricultural pesticides. Nowadays, photo-stable pyrethroids are emerging as the predominant insecticides for vector control. In fact, photo-stable pyrethroids comprise 40% of the insecticides used annually on a global level for indoor residual spraying against malaria vectors and 100% of the WHO-recommended insecticides for the treatment of mosquito nets. The exception is the use of dichlorodiphenyltrichloroethane (DDT) in African countries [2].

Pyrethroid resistance to photo-stable pyrethroids is envisioned to be a major problem for the vector control program since, at present, there are no chemical substitutes for pyrethroids. Moreover, cross-resistance to knockdown agents is the most serious concern. Since this is a global phenomenon, we have started to monitor the distribution of mosquito resistance to pyrethroids. The first pilot study was carried out in Vietnam. In the course of the National Dengue Control Program in Vietnam, the ecological differences between two dengue vectors, namely, *Aedes aegypti* (L.) and *Aedes albopictus* (Skuse), that are of different geographical origins have become a subject of discussion. The ecological differences in the vectors may be the reason for the differences in the epidemics occurring nationwide. In order to examine the nationwide distribution of the two species and pyrethroid resistance, used tires, one of the main breeding grounds of the two species, were targeted for larval collection. Used tires were suitable targets for our study as a standard breeding sites because they were extensively and commonly distributed along roads in Vietnam. In the present paper, distribution analysis of pyrethroid-susceptibility of the larvae of three major mosquito species, namely, *Ae. aegypti*, *Ae. albopictus*, and *Culex quinquefasciatus* (Say), that were collected from used tires located along the national road in Vietnam was carried out. The relationships between pyrethroid susceptibility and the geographical factors and province based annual pyrethroid use are also discussed.

## Materials and Methods

### Collection of mosquito larvae from used tires

**Figure 1:**
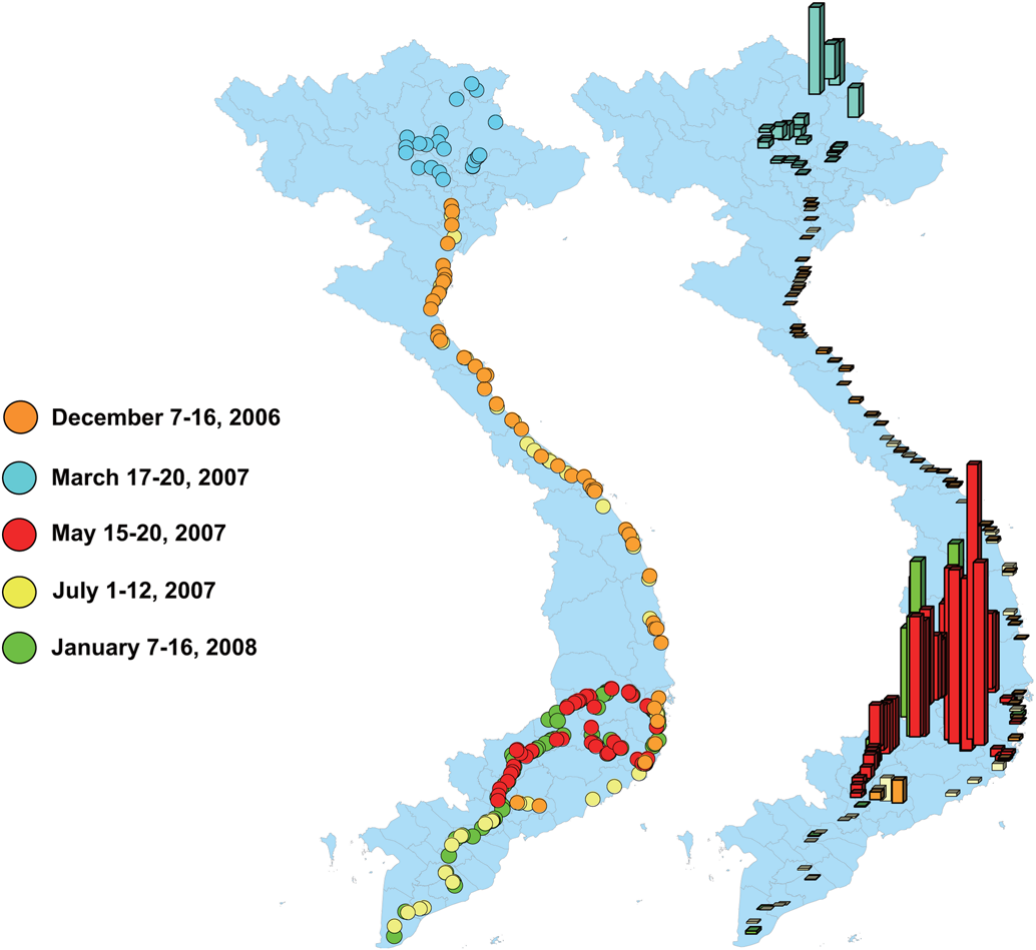
The date and location of the mosquito collection from used tires in Vietnam. The bars in the right map indicate the relative altitude at each collection point (The highest bar indicates 1563 m).

We periodically drove along the national road from the north end to the Mekong Delta in Vietnam (7–16 December 2006; 17–20 March, 15–20 May, and 1–12 July 2007; and 7–16 January 2008) and collected mosquito larvae from used tires (Figure 1). Whenever we encountered the used tires, most of which were found along the periphery of repair shops, while driving along a systematically determined route, the geographical position (with a global positioning system [GPS]), number of tires, presence of water in the tires, existence of mosquito larvae in the tires, and environmental characteristics such as land with vegetation within a 25-m radius and distribution of houses along the road were recorded. Mosquito larvae were collected from tires with larvae (6–20 tires per site) by netting (5 times per tire). The collected larvae, except those used for the susceptibility test, were placed in 1.5 ml plastic vials containing ethanol solution for identification at a later time.

### Simplified knockdown bioassay using the mosquito larvae

The bioassay for the detection of knockdown susceptibility was carried out on the day of collection by using the mosquito larvae obtained from the collection sites from where we could procure an adequate number of insects. The larvae collected from each collection site were briefly identified on the day of collection, and fourth instar larvae of the *Stegomyia* group, which occasionally comprised a mixed population of *Ae. aegypti* and *Ae. albopictus*, and *Cx. quinquefasciatus* individuals, were used for the susceptibility test. Each larva was placed in a glass vial with 20 ml of water. An emulsifiable concentrate of 90% *d*-T_80_-allethrin was diluted with water to obtain a 250 ppm solution. After releasing the larva, 32 or 8 microliters of the solution was added in each vial to obtain a concentration of 0.4 and 0.1 ppm, respectively. Twenty larvae from each site were used for each concentration regime. Knockdown of the larvae was observed for 30 min. Larvae that sank to the bottom of the glass vial and could not swim, float, or were paralyzed were judged as knocked down larvae; the time to knockdown was recorded for each larva. After the test, each larva was placed in a 1.5 ml plastic vial containing ethanol solution for identification at a later time. After identification, individual knockdown data were summarized for each mosquito species (*Ae. aegypti*, *Ae. albopictus*, and *Cx. quinquefasciatus*). The median knockdown times (KT_50_s), i.e., the time required for 50% knockdown, were scored according to the 6 following categories: 1, <5 min; 2, 5–10 min; 3, 10–15 min; 4, 15–20 min; 5, 20–30 min; and 6, >30 min. The susceptibility index was calculated as the product of the scores at 0.1 and 0.4 ppm. Thus, mosquitoes with susceptibility index of 1 were considered to be the most susceptible and those with susceptibility index of 36 were considered to be the least susceptible to *d*-allethrin.

### Knockdown test using adult mosquitoes

In order to confirm the validity of adopting a simplified larval bioassay as a substitute method for determining adult knockdown susceptibility, we performed a knockdown test using several adult colonies of *Ae. aegypti* with known larval susceptibility indices. Four *Ae. aegypti* colonies collected in the field in Vietnam (Ho Chi Minh city, Ben Tre, Ben Luc, Long An; all colonies were collected in 2007) and 1 standard colony (transferred from Sumitomo Chemical Co. Ltd., Hyogo, Japan) that had been maintained in a laboratory after collection were used in the bioassay. A mosquito coil containing 0.3% *d*-T_80_-allethrin was cut into pieces of 0.5 g. A piece of the coil was ignited at both sides and burned completely in a glass chamber (70×70×70 cm) that contained a small battery-operated fan for moving the air. Immediately after the mosquito coil burned out, 3 to 5 day old 20 blood-unfed female mosquitoes were released into the chamber, and their knockdown was observed for 20 min. The test was performed in triplicate. The KT_50_ values were calculated by Bliss' probit method [3].

### Statistical analysis

Univariate analysis was performed to analyze the effects of several factors on susceptibility. Latitude at each collection points were used as the geographical factor. Province based annual pyrethroid uses for malaria control in 1998–2002 (National Institute of Malariology, Parasitology and Entomology, Vietnam, unpublished data) were also used for the analysis. Province-based data were transformed into a raster image using the spline function with ArcGIS 9.2 (ESRI Japan Corp.) to obtain extrapolated values at the collection points. The extrapolated values were log transformed and the susceptibility indices were categorized into two levels, ≥36 and <36, for the statistical analysis using JMP 7.0 (SAS Institute Inc.).

## Results

### Geographical distribution of mosquito species

**Figure 2:**
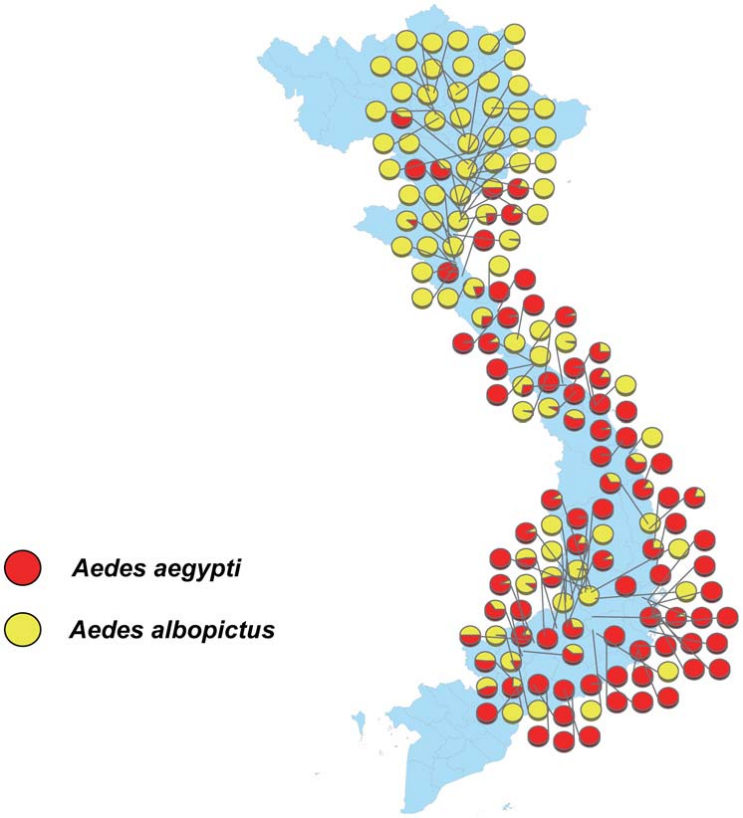
Species composition of the mosquito larvae collected from used tires in Vietnam (Higa et al. unpublished data).

Larvae were collected from used tires along roads in Vietnam from 527 collection points throughout the collection surveillance. Used tires were extensively and commonly distributed. Collection sites were categorized into 5 areas: (1) northern mountainous area (172–506 m in height), (2) northern plain area, (3) eastern coastal area, (4) southern mountainous area (103–1563 m in height), and (5) southern wetlands. Among the 19188 tires surveyed in total during the investigation, 4757 were examined and 2468 contained water (51.9%); 852 contained mosquitoes (34.5%), and of these 653 contained dengue vector mosquitoes (*Ae. aegypti* and *Ae. albopictus*) (26.5%). In total, 8771 *Ae. aegypti*, 5916 *Ae. albopictus*, and 11356 *Cx. quinquefasciatus* larvae were collected. In the northern part of Vietnam, *Ae. albopictus* was dominant, and this dominance gradually reduced toward the south. *Ae. aegypti* was dominant in the southern part. In the eastern coastal areas of the southern part, almost 100% of the *Aedes* larvae collected were *Ae. aegypti*. However, in the mountainous areas, the number of *Ae. albopictus* increased, suggesting that the distribution of the 2 species is determined by different environmental gradients (Figure 2) [4].

### Relationships between the susceptibilities of adult and larval *Ae. aegypti* to *d*-allethrin

**Figure 3:**
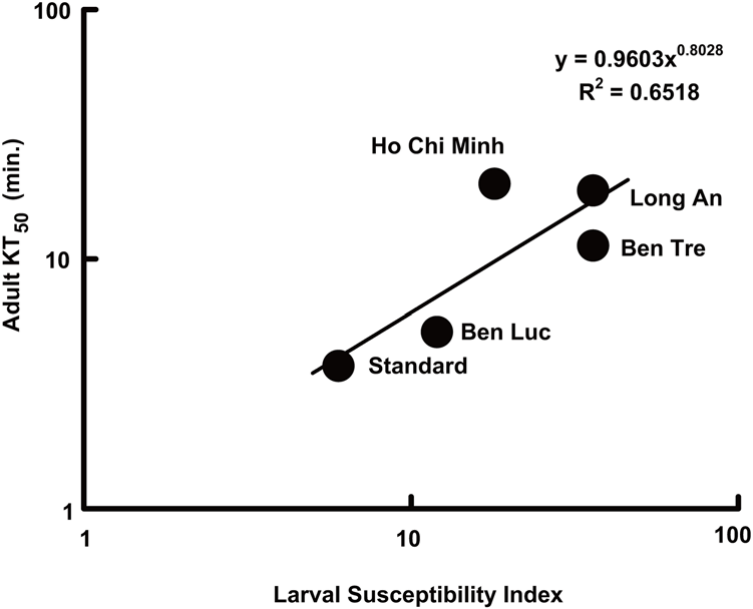
Correlation between the larval susceptibility index and the adult KT_50_ of *Ae. aegypti* collected from several places in Vietnam. Each plot indicates the location from where a mosquito colony was collected. The adult KT_50_ was calculated by the knockdown test by burning 0.5 g of mosquito coil containing 0.3% (w/w) *d*-T_80_-allethrin in a glass chamber (70×70×70 cm).

Figure 3 shows the correlation between the larval susceptibility index and the adult KT_50_ of the same colony of *Ae. aegypti* for samples collected from several places in Vietnam. The adult KT_50_ for the standard strain, which showed normal susceptibility to insecticides was 3.74 min, and its larval susceptibility index was 6. In contrast to the standard strain, the field-collected strains showed higher KT_50_s and larval susceptibility indices. Further, a relatively high correlation was observed between the knockdown susceptibility of larvae and that of adults, indicating that the present simplified knockdown test is a good reflection of adult susceptibility.

### Susceptibility distribution of mosquito larvae collected from used tires in Vietnam

**Figure 4:**
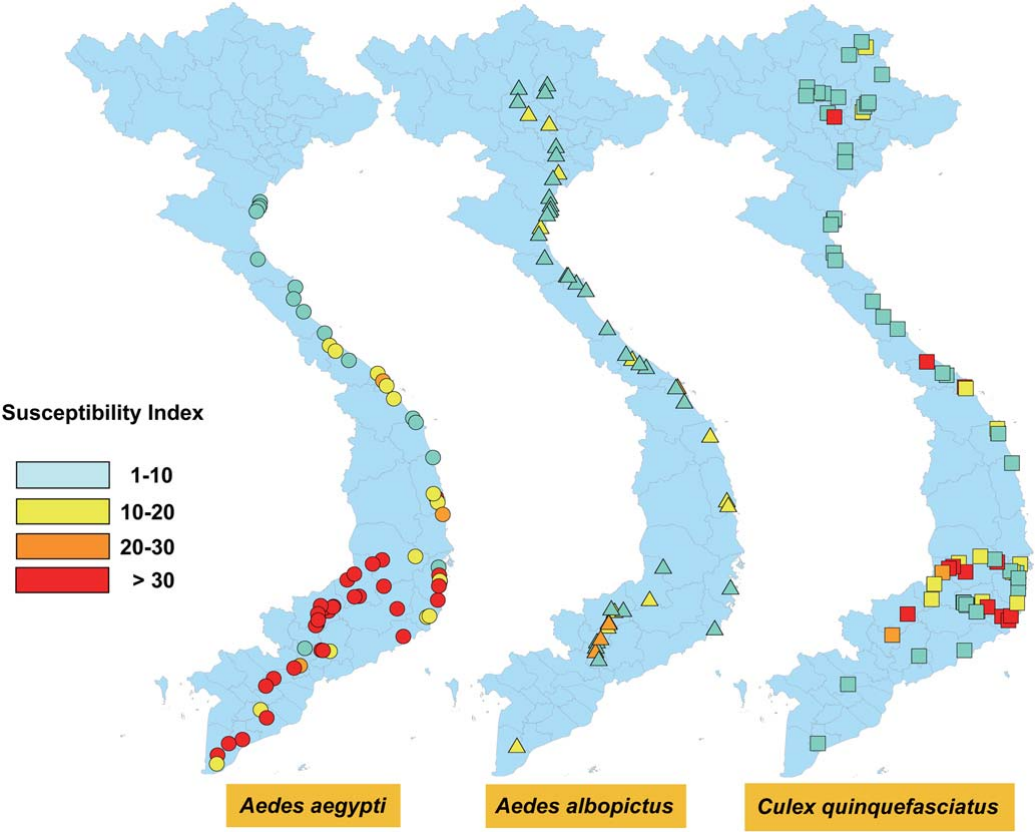
Susceptibility distribution of mosquito larvae collected from used tires in Vietnam.

Distribution of susceptibility indices for *Ae. aegypti*, *Ae. albopictus*, and *Cx. quinquefasciatus* are shown in Figure 4. The number of points where the susceptibility tests were carried out was 67 for *Ae. aegypti*, 50 for *Ae. albopictus*, and 73 for *Cx. quinquefasciatus*. The average susceptibility index and the proportion of mosquitoes with susceptibility indices greater than 20 was 24.9 and 59.7% for *Ae. aegypti*, 8.48 and 8.0% for *Ae. albopictus*, and 12.6 and 21.9% for *Cx. quinquefasciatus*, respectively. When compared with the other species, reduction in susceptibility was most prominent in *Ae. aegypti* (analysis of variance [ANOVA], P<0.0001; Tukey's HSD test, P  =  0.05). In contrast, the reduction in the susceptibility of *Ae. albopictus* and *Cx. quinquefasciatus* was moderate. The susceptibility of the abovementioned species to *d*-allethrin was lower in the southern part (<13°N) than in the northern part (>13°N) of Vietnam. For *Ae. aegypti*, univariate analysis showed significant increases in the susceptibility indices with decrease in the latitude of the collection points (χ^2^  =  36.7, p<0.0001). In contrast, there was no significant correlation between the susceptibility indices and latitude of collection points in *Ae. albopictus* (χ^2^  =  2.57, p  =  0.109) and *Cx. quinquefasciatus* (χ^2^  =  2.43, p  =  0.119). In *Ae. aegypti*, significant correlation between the susceptibility indices and the sum of annual pyrethroid use for malaria control during 1998 and 2002 (χ^2^  =  5.95, p  =  0.0147) was also observed. No significant correlations were seen between the susceptibility indices and the sum of annual pyrethroid use for malaria control in *Ae. albopictus* (χ^2^  =  0.945, p  =  0.331) and *Cx. quinquefasciatus* (χ^2^  =  0.224, p  =  0.636).

## Discussion

The most plausible procedure for investigating the knockdown susceptibility of mosquitoes to pyrethroids is to acquire an adequate number of field-collected female adults or laboratory-reared colonies by rearing field-collected larvae and to carry out a knockdown bioassay with the adults and the actual pyrethroid formulation, such as a mosquito coil, in a laboratory. However, it was impossible for us to follow this procedure since 190 field-collected larval samples were acquired in the present study. Susceptibility tests were, therefore, performed on the day of collection according to a simplified protocol by using the fourth instar larvae. In many cases, the adult susceptibility scores correlated with the larval susceptibility scores [5],[6]. This is, however, not always true for all cases since mosquitoes might develop different resistant mechanisms with different metabolic pathways in the larval and adult stages. In the present study, the authors focused on knockdown that might not be chiefly dependent on the enhancement of metabolic activity but may be dependent on the nervous insensitivity controlled by the *kdr* gene. The simple bioassay with mosquito larvae that is presented in this paper might be a convenient and cost-effective method for evaluating mosquito knockdown resistance in the field.

**Figure 5:**
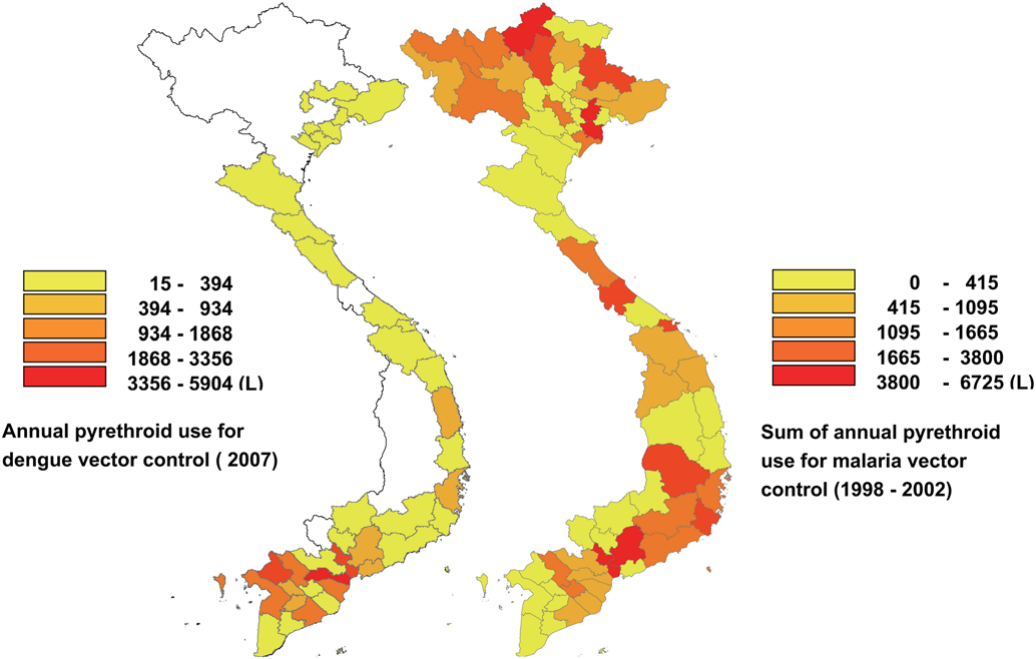
Annual pyrethroid use for dengue vector control in 2007 and the sum of annual pyrethroid use (1998–2002) for malaria vector control in Vietnam. The blanks in the map indicate that no data were available.

The present paper reports some interesting points concerning the distribution of pyrethroid resistance among mosquitoes in Vietnam. The most prominent observation is that the susceptibility of *Ae. aegypti* to *d*-allethrin decreased significantly with decrease in the latitude of the collection points. Vu et al. reported similar tendency in pyrethroid susceptibility in *Ae. aegypti* in Vietnam [7]. The authors conducted WHO standard bioassay using adult *Ae. aegypti* collected in 22 places in 11 provinces and cities in four different regions of Vietnam and found that the mosquitoes were susceptible to pyrethroids in many places in the North and Centre regions but they were resistant in the South and Central Highlands in Vietnam. They concluded this discrepancy in pyrethroid susceptibility in different regions to be due to the longer and extended use of pyrethroids in malaria and dengue fever control programs and in agriculture in the Southern and Central Highlands. Actually, a lot of pyrethroids have been treated as residual treatment inside houses and pyrethroid-impregnated bed nets for malaria control (Figure 5) as a part of the National Malaria Control Program [8],[9],[10]. The pyrethroid use for malaria control seems to be important factor in developing pyrethroid resistance in *Ae. aegypti* in highland region, since the DF/DHF cases are not serious [11] and forest malaria continues to be endemic [12],[13],[14] in highland region as compared to the other regions and consequently the amount of pyrethroid treatment for dengue vector control in highland region is lower than the other regions (Figure 5). The pyrethroid treatment for malaria vector control appears to have been intensively conducted in the interior and along the periphery of human habitation areas, where incidentally, the breeding and resting sites of *Ae. aegypti* are located. This might account for the strong selection pressure toward *Ae. aegypti* and not so much toward *Ae. albopictus* since Asian *Ae. aegypti* is generally a domestic and endophagic that has a greater preference for indoor breeding than *Ae. albopictus* [15],[16],[17],[18],[19]. Several papers report the pyrethroid resistance of both Asian *Ae. aegypti* and *Ae. albopictus*. Most of them report that *Ae. aegypti* had higher pyrethroid resistance than *Ae. albopictus*, indicating pyrethroid resistance was affected by ecological differences in mosquitoes [20],[21],[22],[23].

In Vietnam, 24 tonnes of DDT was used for residual treatment against malaria vectors in 1993 and 1994. However, since the abandoning of DDT sprays in 1995, only pyrethroids (residual spraying of λ-cyhalothrin and α-cypermethrin and occasionally deltamethrin, and permethrin-impregnated bed nets) have been extensively used in large amounts, unlike in the other Asian countries [2],[10]. Although details regarding the amount of insecticides used for dengue control in Vietnam have not been published, 21,000 liters of photo-stable pyrethroid formulations such as λ-cyhalothrin, deltamethrin, and permethrin was reported to be used for dengue control in 20 southern provinces in 2007 [24]. The extensive use of photo-stable pyrethroids, therefore, seems to have been very common in southern Vietnam.

Insecticides still provide the most promising countermeasures for controlling malaria, dengue hemorrhagic fever (DHF), and other arthropod-borne diseases. On an average, at the global level, 547 tones of DDT, 39 tones of organophosphates, 23 tones of carbamates, and 41 tones of pyrethroids are used as active ingredients annually for indoor residual spraying against malaria vectors [2]. The average total amount of pyrethroids used annually as active ingredients between 2003 and 2005 at the global level was 161 tones, which is 16% of the total insecticide consumption and 36% of the total insecticide consumption if the amount of DDT, which is exclusively used in African countries, is excluded. Among pyrethroids that are used for vector control, 98.7% comprise photo-stable pyrethroids such as α-cypermethrin, cypermethrin, bifenthrin, cyfluthrin, deltamethrin, etofenprox, λ-cyhalothrin, and permethrin.

On the other hand, the most popular and long-standing formulations using pyrethroids are mosquito coils, mosquito mats, and liquid vaporizers. Pyrethroids belonging to the knockdown agent group, such as allethrin, pyrethrin, and prallethrin, are used in these formulations. In particular, *d*-allethrin still continues to be used in these types of formulations. The use of pyrethroids for preventing mosquito bites as a “spatial repellent” [25],[26],[27],[28],[29],[30],[31],[32],[33],[34] is believed to be biorational since mosquitoes develop minimum physiological resistance, since it does not kill the affected insects and causes no selection pressure on insect populations. Several factors are believed to play major roles in inducing pyrethroid resistance in mosquitoes. The most serious factor is the uncontrolled use of photo-stable pyrethroids. Photo-stable pyrethroids persist on substrates such as wall and floor surfaces for long periods and hence continue to kill insects that make contact with these substrates. This induces a strong selection pressure on the insect population resulting in a population of resistant offspring. Ultra-low volume (ULV) space sprays are commonly applied for the emergency control of dengue vectors in urban areas. Space sprays by themselves do not induce a very strong selection pressure; however, uncontrolled treatment at high frequency facilitates the development of resistance. In the past, the use of pyrethroids in aqueous environments was impossible since pyrethroids are highly toxic to aqueous organisms. However, recently, a new pyrethroid with low fish toxicity has been commercially produced and is widely used in aqueous environments such as paddy fields. This might cause considerable selection pressure on the mosquito larvae distributed in such environments. The discovery of the phenoxybenzyl alcohol moiety accelerated the development of photo-stable pyrethroids that could be used for agricultural purposes. These “second generation” pyrethroids have been used worldwide as good vector control agents with various application techniques such as residual spraying, ULV spraying, and bed net treatment (long-lasting insecticide-treated net [LLITN]). However, photo-stable and highly effective pyrethroids might accelerate the development of pyrethroid resistance in mosquito populations. The most serious problem is that resistance to a single pyrethroid causes cross-resistance to all other pyrethroids, including knockdown agents. In fact, many reports concerning pyrethroid resistance have emerged after the successful application of pyrethroids as vector control agents [35]. Therefore, the uncontrolled use of such pyrethroids might lead to the end of the golden age of pyrethroids.

Humans have invented insecticides to ensure comfort and to achieve ideal conditions. Good insecticides, therefore, should be as effective as possible so that the abovementioned goals are realized. However, the development and manufacturing costs of insecticides should be as low as possible. It is, therefore, our duty to use insecticides in the most effective and prudent manner possible in order to maintain their effectiveness and sustain their use. In order to effectively manage pyrethroid resistance, the establishment of a feasible insecticide management system and a regular monitoring system of pyrethroid susceptibility will be essential. Moreover, it is expected that the use of photo-unstable knockdown agents as spatial repellents, which effectively interfere with disease transmission without causing any selection pressure to insect populations, will be reconsidered.
